# Early postoperative endoscopic score can predict the long-term endoscopic outcomes in eosinophilic chronic rhinosinusitis (ECRS) patients

**DOI:** 10.1016/j.bjorl.2021.12.003

**Published:** 2022-01-05

**Authors:** Kosuke Akiyama, Yasushi Samukawa, Hiroshi Hoshikawa

**Affiliations:** Kagawa University, Faculty of Medicine, Department of Otolaryngology, Kita-gun, Miki-cho, Japan

**Keywords:** Surgery, Sinusitis, Chronic disease, Eosinophils, Endoscopy

## Abstract

**Objective:**

Eosinophilic Chronic Rhinosinusitis (ECRS) is refractory and recurrent, requiring long-term follow-up after Endoscopic Sinus Surgery (ESS). Endoscopic evaluation is common during postoperative assessment, but how the findings vary over time in postoperative ECRS patients who were treated by the recent standardized management is unclear. We assessed the long-term change in postoperative endoscopic score in ECRS patients using a novel endoscopic scoring system (Escore).

**Methods:**

This retrospective study included 80 ECRS patients who underwent full-house ESS and were followed for longer than 1-year. Endoscopic procedures were repeated at every follow-up visit and postoperative Escores were assessed from 3-months up to 5-years (median follow-up period was 3-years).

**Results:**

The Escore did not significantly change from that at 3-months (3m-Escore). The Escore at the final observation point (f-Escore) among 80 patients was 9.2 ± 5.6 and there was no significant difference from the 3m-Escore (8.5 ± 4.1, *p* = 0.363). Twenty-one patients (21/80, 26.3%) were considered to have endoscopically uncontrolled ECRS at their final observation points and the 3m-Escore was identified as an independent predictive factor by multivariate logistic regression analysis. The 3m-Escore cut-off value that was able to predict endoscopically uncontrolled ECRS after long-term follow-up was ≥12.

**Conclusion:**

We demonstrated the long-term endoscopic outcomes after full-house ESS and continuous outpatient treatment. Early endoscopic findings (3m-Escore) were a potential predictive factor for the later endoscopic outcome, and a 3m-Escore of 12 or higher may be an indicator of the poor long-term prognosis of sinus mucosa.

**Level of evidence:**

4.

## Highlights

• Eosinophilic chronic rhinosinusitis is a poor prognosis and refractory disease.
• We assessed the long-term endoscopic outcomes after surgery and following treatment.
• Early postoperative endoscopic findings are a predictive factor for the later outcomes.
• Higher endoscopic score of 3 months post operation indicates poor long-term prognosis.

## Introduction

Eosinophilic Chronic Rhinosinusitis (ECRS) is classified in the group of CRS with Nasal Polyps (CRSwNP), which was reported in detail in 2015 in Japan.^1^ The diagnostic criteria were defined, and the factors associated with a poor prognosis and refractory disease were narrowed down more than those for CRSwNP in Western countries.^1^, ^2^ Some preservative therapies are not sufficiently effective and Endoscopic Sinus Surgery (ESS) is frequently required for ECRS patients. On the other hands, a multicenter, extensive survey called “The Japanese Epidemiological Survey of Refractory Eosinophilic Chronic Rhinosinusitis (JESREC) study” reported a high recurrence rate, with more than half of the ECRS patients developing recurrence within 5-years after ESS.^1^ Therefore, long-term follow-up and appropriate evaluations are essential in patients with ECRS after surgical treatments.

Evaluation of symptom severity and QOL improvement are important as subjective measures of postoperative sinonasal diseases,^3^, ^4^ but endoscopic evaluation in the sinus mucosa is also valuable. Although, radiological examination is superior for detailed assessments, it is difficult to perform repeatedly due to radiation exposure and medical cost. ECRS is an intractable disease, and life-long follow-up and treatments are required after surgery. Endoscopic examination can be performed easily and repeatedly, making it more suitable for longitudinal postoperative evaluation. In addition, indications for additional treatments, including re-operation or biologics, in patients with a poor prognosis usually consider the endoscopic findings.^5^ The standard surgical methods and effective postoperative treatments were recently established in Japan.^2^, ^6^ However, how endoscopic findings vary over time in postoperative ECRS patients who received such treatments remains unclear. We report the long-term transition in the postoperative endoscopic score using a novel endoscopic scoring system (Escore).^7^

## Methods

### Study population

This retrospective study was conducted between May 2014 and December 2019. One hundred and fifteen consecutive patients who were diagnosed with ECRS and underwent bilateral ESS at Faculty of Medicine, Kagawa University were enrolled in the retrospective study. Eighty adult patients who were followed for at least 1-year were included in the present study. Almost all excluded patients did not reach the 1-year observation period because they were followed-up at another hospital, and there was no patient who received revision surgery or biologics within 1-year after surgery. A definitive diagnosis of ECRS was reached according to the JESREC diagnostic criteria based on a JESREC score of higher than 11 and the presence of more than 70 eosinophils per high-power field in an average of three eosinophil-rich regions.^1^ Exclusion criteria were as follows: age younger than 20-years and patients who were using continuous systemic steroids or biologics at the time of surgery. This study design was approved by the Institutional Review Board, Faculty of Medicine, Kagawa University (approval nº 2020-201).

### Surgical procedure and peri-/postoperative interventions

Bilateral ESS was performed on all patients under general anesthesia. ESS was performed based on the concept of complete resection of the sinus wall and complete opening of all sinuses to make a single sinus (full-house ESS).^8^ If polyps or edematous mucosa were present in the Olfactory Cleft (OC), they were shaved with a microdebrider. Septoplasty, submucosal inferior turbinectomy, and bilateral or unilateral submucosal middle turbinectomy were performed where indicated.^9^ Nasal packing was inserted into the middle meatus and an absorptive gelatin sponge was placed at the OC if the surgical treatment was performed there. All surgeries were performed by two otolaryngologists (the first and second authors). Oral Prednisolone (PSL) at a dose of 15–25 mg per day (0.3 mg/kg as a guide) with gradual tapering was administered from several days before to 2–3 weeks after surgery.^10^ Low-dose long-term clarithromycin macrolide therapy was continued for 3-months after surgery. All patients were followed up at 1- to 4-week intervals until 3-months after surgery to frequently remove secretions, blood crusts, and fibrin under endoscopy, and continuous long-term follow-up was performed at intervals of 1–3 months thereafter. A topical steroid spray was prescribed before and after surgery, and daily saline irrigation was performed by patients. Short bursts of oral PSL therapy (10–20 mg/day) for several days or nasal drop of betamethasone sodium for 1- to 2-weeks were added in the event of acute exacerbation. Antibiotics (amoxicillin or levofloxacin) were administered for a week when bacterial infection with purulent nasal discharge developed.

### Evaluations

JESREC scores were assessed according to the JESREC scoring criteria based on individual CT, nasal findings, and blood sample tests (maximum score, 17).^1^ Patients underwent blood sampling tests, including allergen specific IgE (house dust mite, pollens, and fungi) at baseline, and patients who had specific symptoms and positive antigen tests (more than 0.7 UA/mL) were diagnosed with allergic rhinitis. The diagnosis of Bronchial Asthma (BA) was made by experienced pulmonologists based on bronchial symptoms and some lung function tests. Patients with a history of respiratory exacerbation after receiving Non-Steroidal Anti-Inflammatory Drugs (NSAIDs) were considered to have NSAID sensitivity. Computed Tomography (CT) was performed preoperatively and 3-months after ESS, and was scored according to the Lund-Mackay CT scoring system (score range between 0 and 24).^11^, ^12^ The results of failure to complete removal of ethmoid cells were measured on 3-month CT by the Residual Ethmoid Cells (REC) score. The scoring was performed according to a previous report as follows: bilateral ethmoid sinuses were each divided into superior-anterior, inferior-anterior, and posterior areas, and the RECs in each area were assessed using a 3-graded scoring system (0, absence of any lamina or <4 mm in thickness; 1, presence of lamina ≥4 mm, but there is no continuity with other lamina; 2, at least 2 laminas exhibit continuity but they form incomplete cells; 3, complete cells remain), and the total bilateral possible score was 18.^6^ Endoscopic scoring was performed using a E scoring system with slight modification.^7^ Each sinus and OC were rated 0–2 (0, normal; 1, partially diseased; 2, completely closed or unobservable), and the total bilateral E score ranged from 0 to 2 4. The score was measured using a 4-mm, 30 ° rigid endoscope. Endoscopy procedures were repeated at every follow-up visit, and all exams were video recorded and saved on the server. The percentage of the total score to the maximum possible score for operated sinuses was rated as the Escore in the original method; however, we simply rated it as points in this study because all sinuses were bilaterally opened in all patients. The Escore at 3-months (3m-Escore), 6-months (6m-Escore), 1 year (1y-Escore), and subsequent years (±2-months) up to 5-years after ESS (2y- to 5y-Escore) were adopted in this study. When patients required continuous systemic steroids or biologics due to the exacerbation of upper/lower respiratory disease, revision surgery, or development of another new entry disease, their evaluations were stopped, and all subsequent observations were excluded from the study. Patients in whom the mucosal condition was poor, it was difficult to observe the middle meatus, and there was no improvement noted at the time of final observation were judged as failure to achieve endoscopic control of the disease (uncontrolled group).

### Correlation with existing scoring systems

The Escore system is a relatively newly proposed endoscopic scoring system, and its correlation with 2 established endoscopic scoring systems, Modified Lund-Kennedy endoscopic (MLK) score and the Perioperative Sinus Endoscopy (POSE) score, was confirmed. The MLK was measured for edema (0; absent, 1; mild, 2; severe), polyps (0; no polyps, 1; polyps in middle meatus, 2; beyond middle meatus), and discharge (0; no, 1; clear, thin discharge, 2; thick, purulent discharge).^13^ The scarring and crusting subscores were subtracted from the original LK score, and the total bilateral possible score was 12. The POSE score was rated based on the degree of 10 parameters (each 0–2 points: middle turbinate, middle meatus, maxillary sinus contents, ethmoid cavity; crusting, mucosal edema, polypoid change, polyposis, and secretions, frontal recess/sinus, and sphenoid sinus) for a bilateral total possible score of 40.^14^

### Statistical analysis

All statistical analyses were performed with EZR (Saitama Medical Center, Jichi Medical University, Saitama, Japan). Differences between the 3m-Escore with the other observation points were assessed using the paired *t*-test. Correlations between the Escore at the final observation point (f-Escore) and 3m-Escore were evaluated by Spearman’s *r*-test. The significance of differences between the uncontrolled group with each dependent variable was screened by univariate analysis, and subsequently the potential predictors (*p* < 0.1 in the univariate analysis) were included in the multivariate regression test and narrowed down by backward stepwise selection to identify independent factors. To derive cut-off values of the 3m-Escore for the prediction of endoscopic long-term outcome, we constructed a ROC curve. Differences in patient characteristics between the groups were assessed using the Student’s *t*-test or Fisher’s exact test. Spearman’s *r*-test was performed to determine potential differences and correlation coefficients between the Escore and other endoscopic scoring systems. Significance was assumed when *p* < 0.05. All statistical studies were supervised by a statistician.

## Results

### Characteristics of patients and long-term course of Escore

**Figure 1:**
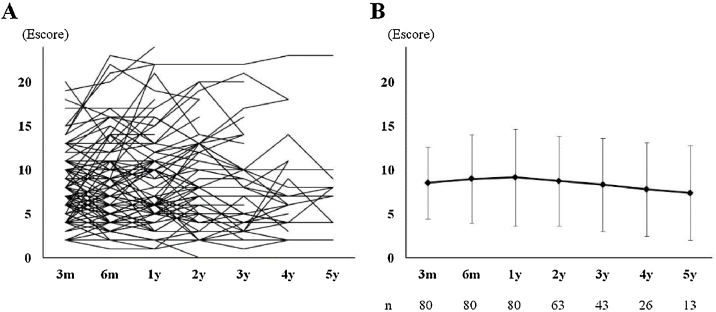
(A) Changes in individual of Escore from 3-months to 5-years postoperatively in 80-patients. (B) Mean change in the Escore from 3-months to 5-years. There was no significant difference between time periods. Error bar shows standard deviation (m, month; y, year).

**Figure 2:**
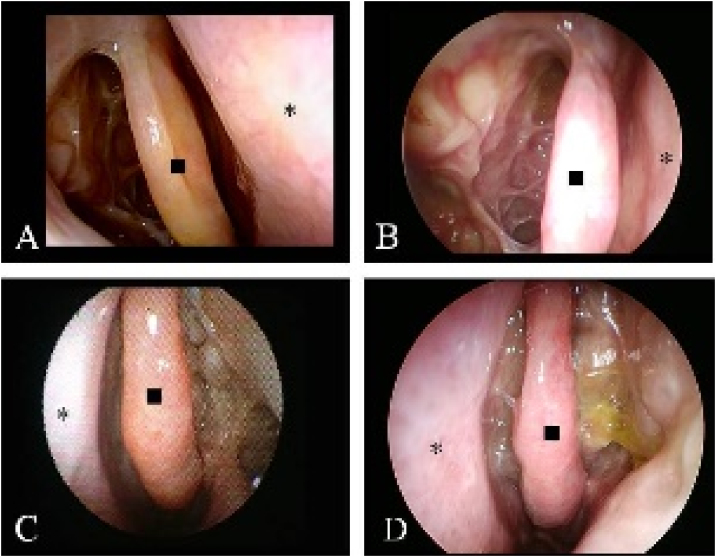
Serial endoscopic records of the nasal cavity in typical cases. Well controlled case: (A) 3-months after surgery, right Escore is 4 (bilateral 7); (B) After 5-years, right Escore is 3 (bilateral 6). Uncontrolled case: (C) 3-months after surgery, right Escore is 8 (bilateral 16); (B) After 4-years, right Escore is 10 (bilateral 21). (◼; middle turbinate, *; nasal septum).

The baseline characteristics are described in Table 1. All patients were observed for longer than 1 year, and subsequent endoscopy evaluations were available for 63, 43, 26, and 13 patients at 2-, 3-, 4-, and 5-years, respectively. Twenty patients were excluded halfway through their observation periods: 7 received biologics, 4 required continued systemic steroid use (3 patients for EGPA and 1 patient for uncontrolled BA), 3 underwent revision surgery, 3 changed their follow-up hospital, and 3 withdrew. The number of patients who required short-burst steroids or antibiotics at least once during their follow-up was 28/80 (35%) and 29/80 (36.5%), respectively. Their average usage counts were 1.8 ± 1.9 times/year and 0.5 ± 0.3 times/years, respectively. All individual Escore changes from 3-months to 5-years postoperatively are described in Fig. 1A. Overall 3m, 6m, 1y, 2y, 3y, 4y, and 5y-Escores were 8.5 ± 4.1, 8.9 ± 5.0, 9.2 ± 5.5, 8.8 ± 5.1, 8.3 ± 5.8, 7.8 ± 3.3, and 7.4 ± 5.2, respectively (Fig. 1B). There was no significant difference between time periods. The f-Escore in 80 patients (median observation period was 3-years) slightly increased from 3-months (mean percent change of score 114 ± 74%), but no significant differences were observed compared with the 3m-Escore (9.2 ± 5.6 vs. 8.5 ± 4.1, *p* = 0.363). Typical endoscopic findings are shown in Fig. 2.

**Table 1:** Characteristics of all 80 patients.

Age (years, mean ± SD)	53.1 ± 10.6
Sex (Male/Female)	29/51
JESREC score (mean ± SD)	14.4 ± 2.3
Allergic rhinitis	35 (43.8%)
Bronchial asthma	60 (75%)
NSAID sensitive	9 (11.3%)
History of sinus surgery	15 (18.8%)
WBC (10^3^/μL, mean ± SD)	6552 ± 1816
Proportion of blood Eos (%)	9.0 ± 5.1
Total IgE (IU/mL, mean ± SD)	477.3 ± 981.2
Preoperative CT score (mean ± SD)	15.4 ± 5.3

JESREC, The Japanese Epidemiological Survey of Refractory Eosinophilic Chronic Rhinosinusitis; NSAID, Non-Steroidal Anti-Inflammatory Drug; WBC, White Blood Cell Count; Eos, Eosinophil.

*Significantly different (*p* < 0.05).

### Relationship between the 3m-Escore and endoscopic long-term outcome

**Figure 3:**
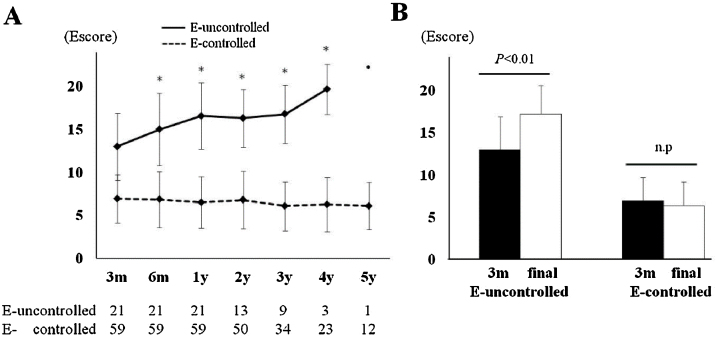
(A) Mean change in the Escore by group. (B) Comparing the mean change in 3m-Escore at the final observation point. Error bar shows the standard deviation. E, Endoscopically; m, month; y, year. *Significantly different from 3m-Escore by paired *t* test (*p* < 0.05).

**Figure 4:**
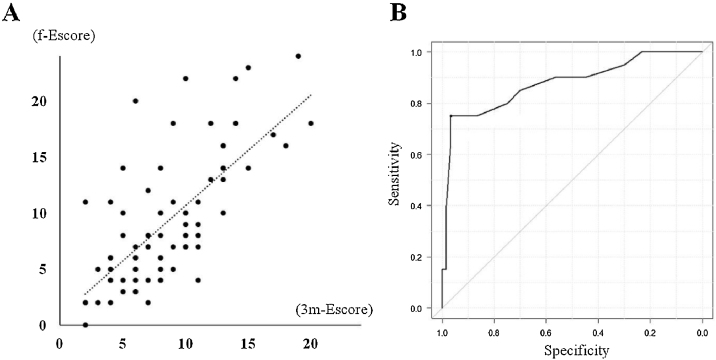
(A) Correlation diagram between 3m-Escore and f-Escore (Spearman's rank correlation coefficient = 0.714, *p* < 0.01). (B) Receiver Operating Characteristics (ROC) curve of 3m-Escore and uncontrolled. The cut-off value of 3m-Escore was defined as 12 (area under the curve = 0.894, 95% CI: 0.8–0.988). 3m-Escore, Escore at 3-months; f-Escore, Escore at the final observation point.

Twenty-one patients (21/80, 26.3%) were considered to have endoscopically uncontrolled ECRS at their final observation points. Subgroup analyses comparing the endoscopically uncontrolled and controlled groups are describe in Table 2. As baseline factors, the presence of NSAID sensitivity and CT score were significantly different, and some long-term evaluation factors, including postoperative CT score, prednisolone usage rate, 3m-Escore, and f-Escore, were significantly higher in the uncontrolled group. Gradual deterioration of the average Escore and a significant difference comparing the 3m-Escore with the f-Escore were observed only in the uncontrolled group (Fig. 3). Multivariate logistic regression analysis was performed to identify independent predictors of a poor endoscopic outcome in the long-term course. Some cofactors (Table 1, REC score, and 3m-Escore) were preliminarily screened for univariate significance. The potential predictors (*p* < 0.1 in the univariate analysis) were included in the multivariate logistic regression analysis and narrowed down by backward stepwise selection. Only the 3m-Escore was significant, with an adjusted odds ratios of 1.72 (95% CI: 1.30–2.27; *p* < 0.01). Therefore, the 3m-Escore was considered to be an independent predictive factor for endoscopic long-term outcome, and it had a strong correlation with the f-Escore (Spearman's rank correlation coefficient = 0.714, *p* < 0.01), (Fig. 4A). To determine the cut-off value for the prediction of endoscopic uncontrolled ECRS, the ROC curve was plotted (Fig. 4B). Lastly, the cut-off value of the 3m-Escore was defined as 12 (area under the curve = 0.894, 95% CI: 0.8–0.988), and the sensitivity and specificity were 75% and 96.7%, respectively. In the present study, 17 patients had a 3m-Escore of 12 or higher and the other 63 patient’s scores were less than 12. Sixteen/seventeen (94.1%) patients whose 3m-Escore was ≥12 had poor endoscopic control of the disease. In contrast, only 5/63 (7.9%) patients with a score <12 was endoscopically uncontrolled at the final observation point.

**Table 2:** Characteristics by each group.

	E-uncontrolled	E-controlled	*p*-value
	(n = 21)	(n = 59)	
Age (years, mean ± SD)	49.5 ± 11.6	54.3 ± 10.1	0.078
Sex (Male/Female)	8/13	21/38	0.943
JESREC score (mean ± SD)	15.3 ± 1.5	14.2 ± 2.5	0.074
Allergic rhinitis	12 (57.1%)	23 (38.9%)	0.202
Bronchial asthma	18 (85.7%)	42 (71.2%)	0.247
NSAID sensitive	5 (23.8%)	4 (6.8%)	0.049^a^
History of sinus surgery	5 (23.8%)	10 (16.9%)	0.523
WBC (10^3^/μL, mean ± SD)	6025 ± 2333	6725 ± 1567	0.132
Proportion of blood Eos, %	9.8 ± 4.4	8.8 ± 5.3	0.429
Total IgE (IU/mL, mean ± SD)	727 ± 1123	390 ± 907	0.201
Preoperative CT score (mean ± SD)	17.9 ± 4.2	14.5 ± 5.3	0.011^a^
3m postoperative CT score (mean ± SD)	9.7 ± 4.6	4.7 ± 3.2	<0.01^a^
REC score (mean ± SD)	2.9 ± 2.9	2.9 ± 2.3	0.958
Use of oral steroids	15 (71.4%)	13 (22%)	<0.01^a^
Use of antibiotics	10 (47.6%)	19 (32.2%)	0.291
3m-Escore (mean ± SD)	13.0 ± 3.9	6.9 ± 2.8	<0.01^a^
f-Escore (mean ± SD)	17.2 ± 3.4	6.4 ± 2.8	<0.01^a^

E, Endoscopically; JESREC, The Japanese Epidemiological Survey of Refractory Eosinophilic Chronic Rhinosinusitis; NSAID, Non-Steroidal Anti-Inflammatory Drug; WBC, White Blood Cell Count; Eos, Eosinophil; REC, Residual Ethmoid Cells; 3m-Escore, Escore at 3-months; f-Escore, Escore at the final observation point.

a Significantly different (*p* < 0.05).

### Correlation with established scoring systems

In the 80 patients, the average MLK scores were 3.8 ± 1.9 and 4.7 ± 2.7 at 3-months and final observation point, respectively, and average POSE scores were 10.4 ± 6.2 and 12.3 ± 8.4 at 3-months and the final observation point, respectively. The Pearson correlation coefficients demonstrated a strong positive correlation between the Escore and 2 established endoscopic scoring systems (Table 3).

**Table 3:** Pearson correlations between Escore and different endoscopic scoring systems.

Correlation coefficient (95% Confidence Interval), n = 80		
	3m-Escore	f-Escore
3m-MLK	0.879 (0.817–0.921)^a^	–
3m-POSE	0.85 (0.775–0.902)^a^	–
f-MLK	–	0.917 (0.874–0.946)^a^
f-POSE	–	0.904 (0.854–0.937)^a^

3m, Each scoring score at 3-months; f, Each scoring score at the final observation point; MLK, Modified Lund-Kennedy endoscopic score; POSE, Perioperative Sinus Endoscopic scoring system.

a Significantly different (*p* < 0.05).

## Discussion

Nasal endoscopy is a common and routine procedure for otolaryngologists to diagnose and assess the postoperative sinonasal mucosal condition. We evaluated the long-term outcomes after ESS in ECRS patients using the Escore in this study. It was previously reported that 20% of ECRS recurs within the first year after ESS and the recurrence rate increases by 10% each year;^1^ therefore, we initially expected the postoperative mucosal condition to gradually deteriorate over time. However, postoperative exacerbation over time was not noted in our study contrary to past reports, and there was no significant difference between the 3m-Escore and following Escore. The surgical concept and following outpatient treatments were standardized to some extent compared with 2015 when the JESREC study was reported, and surgical outcomes were considered to have improved accordingly. As functional ESS is insufficient for ECRS, radical ESS to prevent residual cells is considered sufficient.^6^, ^8^ Ethmoid cells or laminas must be removed thoroughly and strictly based on Okushi’s report that a REC score of 4 or higher is a risk factor for postoperative recurrence.^6^ Frequent removal of fibrin and blood crust during the perioperative period is also important to prevent adhesion on the middle meatus or OC, and it is associated with early improvement of sinus ventilation and ciliated cell regeneration.^15^ In addition, a well-controlled lower respiratory condition is essential to maintain well controlled sinuses mucosa, thus comprehensive treatment on the upper and lower airways is desirable.^16^ In the present study, sufficient surgery may have been achieved in the majority of participants because our average REC score was 3.0 ± 2.5. In addition, we performed frequent outpatient treatment and management of BAs was carried out in collaboration with pulmonologists. Thus, exacerbation over time may be suppressed to some degree by appropriate surgery and continuous management.

On the other hand, a certain number of refractory patients will have a poor prognosis despite such sufficient surgery and treatment. Twenty-one/eighty patients (26.3%) were considered to be uncontrolled endoscopically in the present study. Their 3m-Esore was 13.0 ± 3.9, which was significantly high (6.9 ± 2.8, *p* < 0.01). This suggested that the condition in patients with poor long-term outcomes was already uncontrolled at a relatively early postoperative phase. Further subgroup analysis revealed gradual deterioration only in the uncontrolled group, whereas the Escore in the controlled group was stable without changes over time (Fig. 3).

Based on our supplementary analysis, the 3m-Escore was a significant predictive factor for the f-Escore, and a 3m-Escore of 12 was considered the cut-off value. Sixteen patients (93%) among 17 whose 3m-Escore was higher than the cut-off value and 5 (8%) among 63 patients whose 3m-Escore was lower than the cut-off became uncontrolled. Several factors, such as NSAID sensitivity, eosinophilia, preoperative CT score, and presence of BA, have been reported as predictors of recurrence or a poor outcome.^1^, ^17^ All of these parameters were higher in the endoscopically uncontrolled group than in the controlled group, especially NSAID sensitivity and preoperative CT score in the present study. In addition to these preoperative parameters, the 3m-Escore is valuable as a postoperative predictor that can evaluate other time points. Based on this study, advanced therapies, including biologics or revision surgery, should be considered in patients with scores above the cut-off value because improvement of their long-term mucosal condition is predicted to be difficult even with outpatient management.

Lastly, we evaluated the correlation between Escore and other established endoscopic scoring methods. The LK scoring system was frequently employed for postoperative endoscopic evaluation in previous reports.^18^, ^19^ It is a convenient and reliable method, but it cannot cover the condition of the sinuses or OCs completely. The POSE scoring system was developed to improve the LK system and was designed to assess postoperative sinonasal cavities.^14^ However, it has several disadvantages in that it relatively complicated and it cannot reflect the OC condition. The Escore is a simple and easy method, and it includes the severity of OCs. The E-score was more specialized in assessing the condition of the sinuses and OCs after surgery; therefore, we consider it one of the most optimal evaluating systems for longitudinal follow-up after ESS in ECRS patients. The Escore was also reported to be positively correlated with CT scores, but the score occasionally diverges from the CT score because accurate evaluation of posterior sinuses is sometimes difficult when large polyps are present and prevent observation of the posterior sinuses. This was a limitation of the endoscopic scoring system and was considered inevitable with any method. A strong correlation between the Escore and MLK/POSE score was demonstrated in the present study, and the presented results were not only specific for the application of the E-score, but also for existing scoring systems for long-course prediction.

This study has several limitations. We were unable to assess the long-term symptom/QOL score or olfactory function test systematically, thus comprehensive evaluation of the Escore with subjective symptoms was lacking. The disease specific QOL or symptom score is not significantly correlated with endoscopic examination.^14^, ^18^ Although olfactory disorder is thought to exacerbate over time after ESS, the degree of disorder may depend on other factors, such as the condition of the olfactory cleft, age, or preoperative function;^20^, ^21^ therefore, the Escore alone cannot account for the change. Although, endoscopic examination is essential as an objective test, long-term evaluation combined with the subjective QOL/symptom score is more desirable. Further comprehensive studies are needed to assess the clinical outcomes.

## Conclusion

This study assessed the long-term endoscopic outcomes after full-house ESS and following continuous outpatient treatment. Early endoscopic findings (3m-Escore) were a potential predictive factor for the later endoscopic outcome and a 3m-Escore of 12 or higher may be an indicator of the poor long-term prognosis of sinus mucosa.

## Conflicts of interest

The authors declare no conflicts of interest.
